# A Cloud-Aware Scalable Architecture for Distributed Edge-Enabled BCI Biosensor System

**DOI:** 10.3390/bios16030157

**Published:** 2026-03-13

**Authors:** Sayantan Ghosh, Raghavan Bhuvanakantham, Padmanabhan Sindhujaa, Purushothaman Bhuvana Harishita, Anand Mohan, Balázs Gulyás, Domokos Máthé, Parasuraman Padmanabhan

**Affiliations:** 1 Department of Biophysics and Radiation Biology, Semmelweis University, 1085 Budapest, Hungary; sayantan7@gmail.com; 2 Department of Integrative Biology, Vellore Institute of Technology, Vellore 632014, India; 3 Cognitive Neuroimaging Centre, Experimental Medicine, Nanyang Technological University, Singapore 636921, Singapore; 4 Lee Kong Chian School of Medicine, Nanyang Technological University, Singapore 636921, Singapore; 5 Faculty of Medicine, PSG Institute of Medical Sciences & Research, Peelamedu, Coimbatore 641004, India; 6 Department of Clinical Neuroscience, Karolinska Institute, 17176 Stockholm, Sweden

**Keywords:** Multimodal Brain–Computer Interface, biosensors, EEG, EMG, data processing, edge computing, cloud analytics, electrophysiological signals, real-time signal analysis, scalable data storage, remote health monitoring, continuous biosignal telemetry

## Abstract

BCI biosensors enable continuous monitoring of neural activity, but existing systems face challenges in scalability, latency, and reliable integration with cloud infrastructure. This work presents a cloud-aware, real-time cognitive grid architecture for multimodal BCI biosensors, validated at the system level through a full physical prototype. The system integrates the BioAmp EXG Pill for signal acquisition with an RP2040 microcontroller for local preprocessing using edge-resident TinyML deployment for on-device feature/inference feasibility coupled with environmental context sensors to augment signal context for downstream analytics talking to the external world via Wi-Fi/4G connectivity. A tiered data pipeline was implemented: SD card buffering for raw signals, Redis for near-real-time streaming, PostgreSQL for structured analytics, and AWS S3 with Glacier for long-term archival. End-to-end validation demonstrated consistent edge-level inference with bounded latency, while cloud-assisted telemetry and analytics exhibited variable transmission and processing delays consistent with cellular connectivity and serverless execution characteristics; packet loss remained below 5%. Visualization was achieved through Python 3.10 using Matplotlib GUI, Grafana 10.2.3 dashboards, and on-device LCD displays. Hybrid deployment strategies—local development, simulated cloud testing, and limited cloud usage for benchmark capture—enabled cost-efficient validation while preserving architectural fidelity and latency observability. The results establish a scalable, modular, and energy-efficient biosensor framework, providing a foundation for advanced analytics and translational BCI applications to be explored in subsequent work, with explicit consideration of both edge-resident TinyML inference and cloud-based machine learning workflows.

## 1. Introduction

The last decade has witnessed a surge in the development of non-invasive brain–computer interfaces (BCIs), driven by the convergence of low-cost biosensor technology, embedded intelligence, and ubiquitous connectivity. As clinical and consumer applications expand, BCI systems are now tasked not only with capturing high-fidelity neural and physiological signals, but also with ensuring real-time analysis, robust cloud integration, and scalable deployment outside traditional laboratory environments. Recent surveys of wireless and wearable BCI technologies highlight both the feasibility of mobile brain monitoring and the persistent engineering constraints that emerge outside controlled environments, particularly artifact sensitivity, variable electrode contact quality, and deployment heterogeneity [1]. In parallel, the maturation of edge computing in healthcare has enabled low-latency preprocessing and anomaly detection closer to the point of acquisition, reducing reliance on continuous high-bandwidth streaming while supporting real-time responsiveness for safety-critical or assistive applications [2]. These evolving demands highlight persistent challenges—latency, artifact robustness, and seamless edge–cloud orchestration—that must be addressed to unlock the full translational potential of portable biosensor platforms. The contribution of this work is a validated edge-to-cloud reference architecture and prototype evaluation that emphasizes auditable deployment constraints (edge resource footprint and bounded timing) and role-separated multimodal acquisition (primary inference vs. quality gating), rather than claiming new physiological biomarkers or clinical detection performance. The following subsections review the background, significance, and research objectives that frame the present work.

### 1.1. Background and Significance

BCIs are designed to establish direct communication pathways between neural activity and external systems, enabling control and monitoring without reliance on peripheral neuromuscular output [3,4]. Within the spectrum of non-invasive modalities, electroencephalography (EEG) has historically dominated due to its millisecond temporal resolution, affordability, and long-standing clinical use [5]. Studies have demonstrated its effectiveness in neuroprosthetic applications and rehabilitation, marking it as the canonical entry point for BCI research [6]. However, reliance on a single modality such as EEG imposes constraints in terms of artifact susceptibility, limited spatial resolution, and reduced robustness outside controlled settings, leading to growing interest in hybrid and multimodal BCI approaches integrating additional electrophysiological or hemodynamic signals [7].

Beyond EEG, other electrophysiological modalities have been widely explored to extend the functional scope and robustness of non-invasive BCIs. Electromyography (EMG) and electrooculography (EOG) provide complementary signals by capturing muscle activity and ocular movements, respectively, enabling reliable intent detection in applications such as prosthetic control, speller systems, and assistive interfaces [8,9,10,11]. Hybrid EEG–EMG and EEG–EOG frameworks have been shown to improve control reliability and reduce ambiguity compared to unimodal EEG systems, particularly in real-world and low-signal-quality settings, where peripheral or ocular cues can reinforce cortical intent decoding [12,13].

A second tier of physiological adjuncts supports these core neural modalities. Electrocardiography (ECG) and galvanic skin response/electrodermal activity (GSR/EDA) are frequently integrated to provide autonomic and affective context, improving resilience in multimodal classifiers [14]. Photoplethysmography (PPG), while not a direct neural measure, offers heart-rate variability features that enrich assessments of stress and fatigue, particularly in IoT-enabled wearable systems [15]. These adjuncts act as contextual layers rather than primary BCI channels, and their utility depends on robust real-world data handling across the acquisition-to-analysis pipeline [16].

Despite advances in sensing modalities, existing systems face persistent challenges in latency, scalability, and seamless integration with distributed cloud infrastructures [17,18]. Current commercial wearables, although accessible, often suffer from noisy signals, limited reliability, and poor suitability for deployment in regulated or high-reliability biosensing contexts [19]. These gaps underscore the need for architectures that combine multimodal signal acquisition with robust edge preprocessing and cloud-aware data pipelines, ensuring both performance and scalability in translational contexts. Nevertheless, we do not claim that individual components are unprecedented; rather, the contribution is the integrated, auditable, role-separated architecture plus prototype validation on constrained edge hardware.

### 1.2. Research Objectives and Scope

At the edge, the system employs lightweight preprocessing and TinyML-based inference [20,21] on an Arduino Nano RP2040 Connect (Arduino S.r.l., Monza, Italy) microcontroller, henceforth referred as Nano Connect or simply RP2040, consistent with recent advances in embedded machine learning for biosignal analytics [22]. This design enables consistent, window-bounded inference suitable for near-real-time BCI interaction, while preserving strict energy and resource constraints typical of wearable platforms. Data are transmitted either via Wi-Fi module of Nano Connect or via ESP32 Vajravegha LTE module (Vajravegha Mobility Pvt Ltd, Thane, India), henceforth referred as Vajravegha to a cloud-based pipeline comprising Redis v7.4.0 for near-real-time buffering, PostgreSQL v.17.7 for structured analytics, and AWS S3 with Glacier for archival storage, reflecting established practices in scalable IoT healthcare infrastructures [23]. This architecture separates time-critical edge inference from cloud-assisted analytics and storage, preserving raw biosignal fidelity while enabling downstream processing at scale. The objectives of this work are twofold:

1. To validate that a fully implemented physical prototype can achieve consistent edge-level inference and robust end-to-end operation interacting with the cloud under realistic connectivity conditions, including bounded packet loss and latency variability.
2. To demonstrate a scalable and modular system architecture that remains cost-efficient through staged deployment strategies, including local development, simulated cloud testing, and targeted cloud benchmarking.

By addressing persistent challenges related to latency variability, scalability, and reliable cloud integration, the proposed architecture establishes a foundation for translational BCI systems and large-scale neuroinformatics workflows [24,25]. Future extensions will explore advanced edge-resident and cloud-based machine learning techniques using electrophysiological datasets aligned with non-invasive BCI modalities, enabling longitudinal analysis and model refinement beyond the scope of the present study.

**Figure 1:**
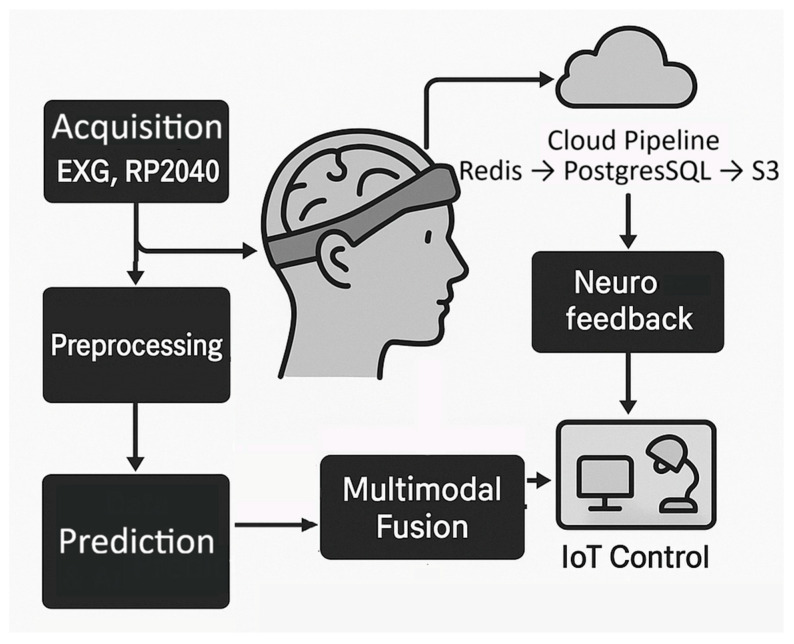
Conceptual overview of the proposed Real-time Cognitive Grid (RCG) system, illustrating the integration of biosignal acquisition, multimodal fusion, remote storage, and feedback/control pathways.

Figure 1 summarizes the proposed architecture, highlighting the flow of biosignal data from acquisition through edge preprocessing to cloud integration. The design emphasizes low-latency, window-bounded edge inference on the RP2040, supported by embedded inference toolchains suitable for resource-constrained TinyML deployment, and tiered cloud storage to ensure both near-real-time responsiveness and scalability in distributed IoT telemetry settings [26,27,28]. Neural and electrophysiological signals are acquired through the BioAmp EXG (Upside Down Labs, New Delhi, India) module (EEG, EMG, EOG, ECG), while complementary modules provide supporting physiological and environmental context including DHT11 (Aosong Electronics Co., Ltd., Guangzhou, China) for temperature/humidity, and MQ-x (MQ-135 (Zhengzhou Winsen Electronics Technology Co., Ltd., Zhengzhou, China) in the prototype, configured to be easily hot-swappable with other MQ sensors) which provides broad-spectrum sensitivity to volatile organic compounds (VOCs), alcohol vapors, ammonia, benzene-type gases, and variations in indoor air quality), all interfaced to enable multimodal acquisition within a portable prototype [29,30,31]. This study aspires to use the RP2040 microcontroller as a novel platform for creating cost effective BCIs. Auxiliary modules are also augmented such as an optical pulse sensor from the MAX3010× family (Maxim Integrated, San Jose, CA, USA) support heart rate and perfusion estimation. These components collectively provide a practical foundation for on-device signal acquisition, embedded analytics, and cloud integration within a controlled research and prototyping setting, while acknowledging that the present hardware is not intended for clinical diagnostics or hazardous-environment certification.

Preprocessing and lightweight classification at the edge reduce noise and extract salient features prior to uplink, separating time-critical inference from cloud-assisted analytics and archival [32]. The resulting streams are transmitted into a tiered data management system for near-real-time buffering and long-term storage [33]. These data are relayed to cloud storage for large-scale analysis and longitudinal retention, while feedback may be returned to the user via neurofeedback or interaction channels depending on the application context. In parallel, detected patterns can be mapped to device-level or application-level control signals, thereby closing the loop between acquisition, contextual interpretation, and actuation.

By distinguishing core electrophysiological modalities from supporting adjuncts, the framework remains multimodal while staying faithful to the prototype’s sensing stack and portability constraints [34]. The modular pipeline supports staged deployment, enabling purely local operation during development while retaining the ability to scale into cloud-assisted analytics for benchmarking and translational studies. To generalize the architecture beyond its prototype implementation, this work introduces the Real-time Cognitive Grid (RCG) framework. RCG is structured into four modular layers: RCG Cortex, responsible for edge-level preprocessing and lightweight TinyML inference; RCG Gateway, which manages secure data uplink through LTE-enabled controllers; RCG Vault, dedicated to structured and archival cloud storage; and RCG Dash, encompassing visualization, dashboards, and alerting mechanisms.

To connect the RCG abstraction to the prototype’s multimodal evidence, we operationalize the RCG layers in terms of modality-specific duties rather than treating all channels as symmetric inference inputs. RCG Cortex executes windowed preprocessing and lightweight edge inference while also emitting signal-quality indicators; the RCG Gateway transports both inference outputs and quality flags over the LTE/Wi-Fi uplink; the RCG Vault persists raw sessions and structured summaries for traceability; and the RCG Dash consumes these streams to render user-visible updates and alerts. This layered separation aligns with established practice in electrophysiological systems where auxiliary channels can be used to quantify reliability and suppress contaminated windows without implying multi-channel classifier parity. Table 1 clarifies the role-separated use of modalities in the present prototype. EMG is the only modality benchmarked for inference performance in this manuscript. EEG and EOG are acquired concurrently to compute lightweight quality indicators that support reliability gating, while ECG is included to demonstrate acquisition feasibility within the same hardware–software pipeline. Accordingly, the multimodal contribution of this work is architectural: concurrent acquisition and reliability-aware edge-to-cloud design, rather than symmetric multimodal classification accuracy. Continuous, multimodal physiological monitoring remains a promising application of the RCG architecture, and future work will include task-driven evaluation under controlled paradigms.

Together, these components establish a portable yet extensible framework that accommodates both multimodal biosignal inputs alongside environmental adjuncts of temperature, humidity, and ambient gas concentrations, providing contextual enrichment and clinical relevance. The conceptual model positions the system as both a validated prototype and a scalable blueprint for broad BCI applications, where modular deployment ensures adaptability across research and translational domains [35]. This manuscript reports an engineering architecture and full-stack prototype evaluation. It does not report a human-subject experimental study or stimulus-driven physiological paradigm intended for population-level inference. Accordingly, the contribution is restricted to system design, acquisition feasibility, dataflow integrity, and quantified system-level performance, while cohort-scale model validation and clinical interpretation are deferred to subsequent work. The prototype supports multiple biosignal modalities (EEG, EMG, EOG, ECG) through standard analog front-end inputs. When deployed in human-subject studies, electrode positioning should follow standard nomenclature (e.g., International 10–20/10–10 for EEG), and surface EMG attachments should follow established clinical placement guidelines. These details are not part of the present engineering evaluation and are mentioned here only as deployment context for future work.

## 2. Related Work

The evolution of BCI systems has been shaped by rapid advances in biosensor hardware, embedded analytics, and cloud-enabled infrastructure. Recent years have witnessed a convergence of engineering and neuroscience, where the deployment of portable, multimodal biosensors—coupled with real-time data pipelines—has transformed the landscape of cognitive state monitoring, neuroprosthetics, and translational neuroinformatics. Parallel progress in edge computing, wireless telemetry, and scalable storage solutions has enabled the development of systems that are not only more responsive and energy-efficient, but also increasingly accessible beyond the confines of laboratory settings. Against this backdrop, the following sections examine prior art spanning biosensor modalities, embedded intelligence, IoT healthcare architectures, and hybrid edge–cloud frameworks, laying the foundation for the unique contributions of the present work.

### 2.1. Overview of Core Non-Invasive BCI Modalities Under Consideration

Non-invasive BCI systems have primarily evolved around a set of well-established sensing modalities, each contributing distinct advantages and limitations. EEG remains the dominant channel, valued for its high temporal resolution and affordability, and it has been widely applied in neuroprosthetics, rehabilitation, and emotion recognition [36,37]. Despite this ubiquity, EEG suffers from limited spatial specificity and susceptibility to artifacts, which has encouraged the development of complementary sensing and hybrid control strategies.

EMG extends BCI capability beyond purely neural activity by enabling intent detection through muscular activation patterns. EMG-based systems are particularly effective for prosthetic control and distributed frameworks that combine motor and neural information [38]. Similarly, EOG leverages ocular activity, including blinks and saccades, to support spellers, cursor control, and multimodal interaction, especially in assistive HCI contexts.

In addition to these core modalities, a second tier of physiological adjuncts has been incorporated into multimodal designs. ECG provides cardiac context relevant for fatigue and stress monitoring, while GSR/EDA offers markers of arousal and attentional modulation. PPG, widely embedded in wearable devices, supplies heart-rate variability indices that serve as contextual correlates and help in artifact removal rather than direct neural inputs [39]. While EDA/GSR signals are supported by the hardware, no controlled stimulation paradigm was performed in this study; signals were acquired and verified for structural integrity under resting conditions. These adjunct signals enrich classification frameworks by embedding autonomic dynamics, but they are rarely used as standalone BCI channels [40,41].

Collectively, the integration of EEG, EMG, EOG, and adjunct signals such as ECG, GSR, and PPG defines the current landscape of portable, non-invasive BCI systems. However, while multimodal designs improve robustness, many existing implementations remain fragmented, often lacking scalable pipelines that reliably bridge acquisition, real-time analytics, and cloud-based storage. This gap underscores the need for unified architectures capable of sustaining both performance benchmarks and translational applicability.

### 2.2. Role of Cloud and Edge Computing in Biosensors

The rapid growth of wearable and portable biosensors has intensified the need for architectures that can deliver low-latency decision support, high-throughput ingestion, and secure longitudinal storage without collapsing under bandwidth, cost, and privacy constraints. Cloud-only pipelines, where continuous raw streams are pushed upstream for every analytic step, can introduce avoidable delays and network bottlenecks, which is especially problematic for time-sensitive BCI-adjacent workflows and closed-loop feedback. Surveys on healthcare edge computing consistently highlight this latency–bandwidth tension as a core driver for pushing computation closer to the signal source. To mitigate these constraints, edge computing has evolved from simple thresholding into a practical layer for signal conditioning, lightweight inference, and event-triggered transmission. TinyML literature emphasizes that local inference reduces dependence on round-trip cloud connectivity and thereby avoids cloud-induced latency for many IoT-class scenarios [42]. In parallel, embedded biosignal work on wearables demonstrates that real-time classification is feasible under tight power budgets when models are quantized and engineered for constrained processors; for instance, TEMPONet-style [43] temporal convolutional pipelines have been implemented on ultra-low-power multicore IoT processors with per-window inference on the order of milliseconds, illustrating what “edge-real-time” can look like in practice for muscular intent streams [44]. The broader implication for biosensor systems is architectural: edge layers should absorb the “always-on” work (denoising, segmentation, compression, early warnings), while the cloud absorbs the “deep and wide” work (population-scale learning, model retraining, cohort analytics, and durable governance) [45]. Meanwhile, cloud infrastructure remains indispensable for (i) scalable, long-term data management and (ii) computationally heavy neuroinformatics workflows. Cloud brainformatics platforms illustrate the value of centralized compute and storage for EEG big-data exploration and analysis, where the cloud is not just a bucket but an ecosystem for reuse, collaboration, and high-performance processing [46]. This becomes more relevant when systems are designed to interoperate with large public datasets and benchmarking ecosystems (e.g., TUH EEG [47] for large-scale clinical EEG, and ADNI [48] for longitudinal neuroimaging and biomarker trajectories), which implicitly demand robust storage, indexing, and access control patterns. From a pipeline-design viewpoint, hybrid edge–cloud stacks commonly adopt a tiered persistence model:

- A fast buffer for “right-now” windows (often an in-memory time-series store);
- A structured store for queryable longitudinal slices;
- An object/archive layer for raw session artifacts and cold retention.

For example, Redis natively supports time-series data handling as a first-class pattern for timestamped sensor points, making it a practical fit for transient buffering and short-horizon aggregation. For longer-horizon analytics, PostgreSQL-based time-series extensions (e.g., TimescaleDB 2.25) are explicitly designed for large volumes of timestamped IoT/sensor readings while retaining SQL semantics, which is useful when you want biomedical metadata joins and audit-friendly schemas. Finally, cloud object storage and archival tiers (such as, Amazon S3 with Glacier classes) formalize cost-aware retention, where “cold” biosignal payloads can be stored with durable, policy-driven retrieval characteristics rather than sitting in expensive hot storage forever. Despite this maturity, the literature still shows a recurring fragmentation: some systems over-optimize edge latency while under-designing interoperability and data governance, whereas others centralize everything in the cloud and inherit avoidable delay, bandwidth waste, and operational cost [49,50]. This motivates architectures that are cloud-aware by design, not cloud-dependent: edge intelligence should be treated as the default for immediacy and resilience, while cloud services should be treated as the amplifier for scale, reproducibility, and translational deployment.

### 2.3. Limitations of Existing Biosensor Frameworks

Although substantial progress has been reported across wearable and portable BCI-oriented sensing, many existing biosensor systems remain constrained by architectural and deployment realities. Consumer-grade wearables and low-cost EEG headsets improve accessibility, yet commonly face practical limitations in signal quality, electrode coverage, and robustness under unconstrained, real-world conditions, which can reduce reliability for translational or clinically adjacent use-cases.

While multimodal and hybrid designs can improve decoding stability, they are frequently validated in controlled settings and remain difficult to scale into reproducible, field-deployable systems without strong attention to calibration, artifact resilience, and operational constraints [51,52,53]. Efforts to integrate biosensors with IoT infrastructures demonstrate clear promise, but many reported frameworks still lack tiered pipelines that simultaneously support low-latency edge responsiveness, structured queryable storage, and cost-aware long-horizon archival. Architectures that over-rely on cloud connectivity inherit bandwidth pressure and latency sensitivity, whereas purely edge-centric designs struggle to support population-scale analytics, cross-session reproducibility, and integration with large longitudinal resources. A further limitation is the absence of consistently adopted design and data standards, which contributes to fragmented deployments and weak interoperability across devices, vendors, and datasets. As a result, benchmarking is often confined to isolated case studies or narrow evaluation settings, rather than scalable validation across heterogeneous acquisition conditions and multi-site data [54,55].

Collectively, these shortcomings motivate unified frameworks that treat portability, multimodal integration, and hybrid edge–cloud intelligence as a single end-to-end system problem, enabling reproducible deployment pathways aligned with translational biosensor applications in healthcare and neuroengineering [56,57,58].

## 3. Materials and Methods

This section documents the methods required to reproduce the engineering prototype evaluation reported in this manuscript. The evaluation is limited to hardware–software integration, acquisition feasibility checks, and quantified system-level performance across repeated end-to-end runs (latency, packet loss, and throughput). No stimulus-driven task paradigm or subject-powered physiological/clinical study is reported in this work. System benchmarking was performed as repeated acquisition-to-cloud runs, where a run is defined as a complete execution of (i) controlled bench input to the BioAmp EXG acquisition chain and/or replay of de-identified biosignal traces, (ii) on-device preprocessing and inference on the RP2040, and (iii) uplink, ingestion, logging, and visualization through the cloud-aware pipeline. Runs were repeated across connectivity conditions (Wi-Fi/LTE) and logging configurations to quantify variability using distributional summaries (mean ± SD and percentiles).

This section details the end-to-end implementation pathway of the proposed biosensor platform, structured to maximize reproducibility across acquisition, embedded processing, and cloud persistence. We first describe the system architecture as a tiered edge–cloud design that assigns latency-critical operations (signal conditioning, event detection, lightweight inference, and bandwidth shaping) to the edge, while reserving scalable functions (long-horizon storage, cohort analytics, visualization, and lifecycle management) for the cloud, consistent with established edge–cloud patterns in healthcare monitoring systems. We then specify the hardware components and instrumentation choices that enable portable deployment, followed by the software stack responsible for orchestration, data transport, and storage. Finally, we formalize the data acquisition and preprocessing pipeline, including artifact-aware handling steps needed for wearable-grade recordings under unconstrained conditions, thereby ensuring that the downstream analytics remain interpretable and comparable across sessions [59,60]. We define a run as a continuous end-to-end execution of acquisition, edge preprocessing/inference, and uplink/ingestion ending in logging/visualization under a fixed configuration. Each run was executed for 10 min and repeated for 15 runs per connectivity condition (Wi-Fi and/or LTE), enabling reporting of variability (mean ± SD and p50/p95) for latency and packet loss.

The deployed network topology comprises (i) an RP2040 edge node interfaced with the BioAmp EXG analog-front-end, (ii) an uplink stage via Wi-Fi or LTE through the Vajravegha gateway, and (iii) a cloud ingest-to-storage-to-visualization path. Latency is therefore reported using explicit measurement boundaries, separating consistent edge compute from network-dependent transport and cloud-side components.

### 3.1. System Architecture

The proposed system was implemented as a physical prototype to validate the feasibility of a cloud-aware multimodal biosensor pipeline. The design followed a layered architecture that combined portable acquisition hardware, embedded edge analytics, and scalable cloud infrastructure [61]. At the acquisition layer, neural and physiological signals were captured using the BioAmp EXG Pill interfaced with an RP2040 microcontroller. This configuration enabled real-time recording of EEG, EMG, and EOG signals, with an option to extend to additional channels for multimodal fusion [62,63]. The RP2040 performed lightweight preprocessing steps including filtering, root mean square (RMS) calculation, and spectral transforms such as fast Fourier transform (FFT).

For local analytics, the RP2040 executed TinyML inference models compiled into C header files, supporting on-device classification of brain states and motor intent [21,29]. In parallel, raw and preprocessed signals were buffered on an SD card for redundancy and offline analysis. The communication layer was managed by on-board Nano Connect Wi-Fi module or ESP32 Vajravegha LTE module, which provided secure data uplink via HTTPS and MQTT protocols. To reduce bandwidth demands, the module transmitted structured packets containing key features and classification outputs, while complete raw signals were stored locally [64,65].

On the cloud side, a tiered pipeline was implemented. Redis served as a low-latency buffer for the first few seconds of signal streams, enabling real-time visualization and anomaly detection [66,67]. PostgreSQL stored structured datasets for analytics and model training, while AWS S3 and Glacier provided long-term archival of session files. Visualization was supported through Python-based graphical interfaces, Grafana dashboards, and an on-device Nokia 5110 LCD for immediate feedback. This modular architecture demonstrated interoperability across acquisition, processing, transmission, and storage layers, ensuring that both prototype validation and translational scalability were addressed.

**Figure 2:**
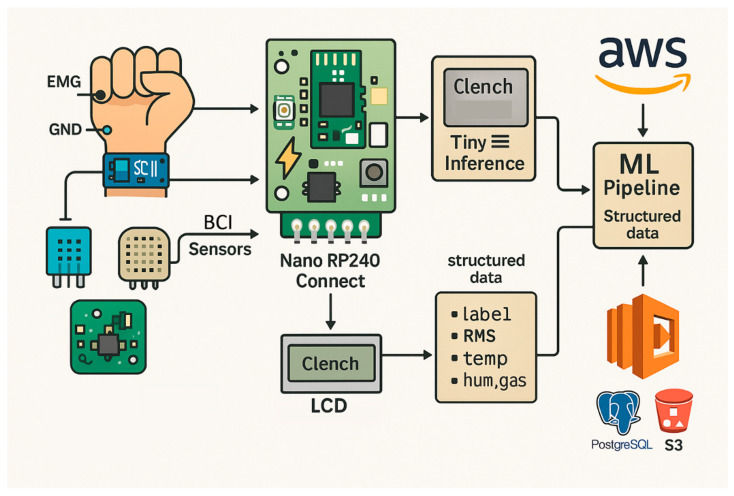
Overview of the edge-to-cloud biosensing system using Arduino Nano RP2040 Connect microcontroller, performing BCI inference and structured data uploads to AWS.

Figure 2 summarizes the layered edge–cloud implementation, showing multimodal acquisition using three BioAmp EXG Pill units configured for EEG, EMG, and EOG, followed by preprocessing and TinyML inference on the RP2040. Environmental sensing (DHT11 for temperature and humidity; MQ-135 for ambient air-quality proxy) is co-sampled as auxiliary context to support deployment-aware interpretation and logging [46]. At the edge, the node provides immediate LCD feedback and SD-card buffering, while structured packets (features, context values, and inference outputs) are forwarded via the available uplink (Wi-Fi or LTE, depending on network availability) to AWS, where PostgreSQL supports structured analytics and S3 provides long-term archival [32,68,69].

The layered workflow can be mapped to the RCG abstraction introduced earlier. In this implementation, RCG Cortex corresponds to the RP2040-based edge preprocessing and TinyML inference; RCG Gateway is realized through Wi-Fi/LTE module (depending on network availability) handling secure transmission; RCG Vault is represented by PostgreSQL, S3, and Glacier providing structured and archival storage; and RCG Dash encompasses Redis-driven streaming, Grafana dashboards, and LCD-based feedback. Embedding the RCG terminology in this build highlights the modularity of the design without introducing redundancy or conceptual overhead.

### 3.2. Hardware Components

The physical prototype was assembled from commercially available microcontrollers, biosignal amplifiers, and environmental sensors. Table 2 summarizes the key components and their roles within the system architecture.

This hardware ensemble enabled simultaneous acquisition of neural, muscular, cardiac, and environmental signals. Each module contributed to the RCG abstraction, ensuring that edge analytics, secure transmission, structured storage, and visualization were consistently represented. All reported results are based on repeated runs using the same prototype hardware configuration; independent device variability and throughput under varying loads were not systematically evaluated in this study.

### 3.3. Software Stack

The software pipeline was implemented as a hybrid of embedded firmware, data processing scripts, and cloud-based services, enabling seamless communication across acquisition, edge analytics, and storage layers. The stack is designed to accommodate additional preprocessing or classification routines and supports rapid integration of new biosignal modalities or context sensors as the hardware platform evolves.

At the device level, the Nano RP2040 Connect was programmed using the Arduino IDE v2.3.2 and PlatformIO v6.1.0 framework, with support libraries for SPI, I^2^C, and analog signal acquisition. Preprocessing routines implemented in C included bandpass filtering, root mean square (RMS) extraction, and fast Fourier transform (FFT). TinyML models were trained in Python using scikit-learn and TensorFlow Lite [70], converted to .tflite format, and compiled into .h header files for integration into the RP2040 firmware [71,72].

For secure uplink, HTTPS and MQTT protocols were employed, supported by embedded SSL/TLS libraries. Structured payloads were encoded in JSON and transmitted to AWS endpoints. A Lambda function parsed the payloads, routing structured entries into PostgreSQL and archiving complete session files into Amazon S3. Lifecycle policies ensured automated migration of older data into Glacier for cost-efficient archival [33,73].

Visualization and monitoring were achieved through Python-based graphical interfaces during development and Grafana dashboards during deployment. Redis served as a low-latency streaming buffer to feed Grafana panels, providing near real-time visualization of incoming biosignals. Local feedback was delivered through the Nokia 5110 LCD, while Python GUI scripts facilitated early-stage debugging and offline inspection.

### 3.4. Data Acquisition and Preprocessing

Biosignals were acquired using the BioAmp EXG Pill connected to surface electrodes. The module supports single-channel acquisition of EEG/EOG by default and can be configured for EMG/ECG via hardware bridging, enabling modality switching while retaining the same analog front-end chain. For EMG feasibility evaluation, the forearm flexor muscle group represents the standard surface placement target for clench and relaxation paradigms; representative EMG activation traces—acquired via controlled bench input and/or replay of de-identified biosignal traces consistent with this placement convention—illustrate that voluntary muscle engagement produces clear amplitude modulation relative to baseline noise, confirming effective signal capture through the acquisition pipeline. For ECG feasibility, a Lead-I style electrode configuration represents the standard reference placement approach for single-channel cardiac monitoring. For EEG feasibility, conservative frontal placements are standard for non-shielded wearable setups, where signal quality is strongly influenced by electrode type, impedance, and motion artifacts. These placement conventions are described here as deployment guidance consistent with standard practice; no dedicated human-subject electrode placement protocol was performed as part of this engineering evaluation.

In the prototype, physiological channels were sampled at 1 kHz. On-device preprocessing uses fixed-length sliding windows of 250 ms with 50% overlap (i.e., 125 ms hop). For each window, the following features were extracted:

- Root-mean-square (RMS) amplitude;
- FFT-derived bandpower over defined frequency bands;
- Statistical moment descriptors.

These features are passed to a lightweight classifier (LDA with a shallow Random Forest ensemble). The deployed classifier artifact uses int8 quantization to ensure bounded memory footprint and predictable execution time. No explicit clinical-grade pre-filters were applied; all filtering and artifact handling represents the minimal processing necessary for embedded feasibility evaluation. Higher-order, cohort-scale artifact modeling is out of scope for this study and will be addressed in future analytics work. On the Nano RP2040 Connect, analogRead() returns 10-bit values by default and can be increased to 12-bit using analogReadResolution(12). The underlying RP2040 platform provides a 12-bit ADC with sufficient headroom for kHz-rate sampling in principle; however, the effective sampling behavior remains implementation-bounded by the firmware loop and buffering strategy. Raw biosignals were logged locally to an SD card for redundancy and offline inspection, while time-stamped feature packets were emitted for near-real-time visualization and cloud ingestion.

Edge preprocessing on the RP2040 applied task-appropriate filtering and feature extraction. For EEG/ECG feasibility, a low-frequency bandpass commonly used in non-invasive BCI preprocessing was adopted (e.g., ~0.5–35/45 Hz) to reduce baseline drift and high-frequency noise while preserving task-relevant rhythms. For surface EMG, bandpass filtering in the ~20–450/500 Hz range was used as a standard artifact/noise suppression practice, optionally combined with notch-based powerline mitigation where required by the recording environment. Time-domain features (e.g., RMS, variance) and short-window spectral features (FFT-derived bandpower summaries) were computed to support lightweight classification under wearable compute constraints.

For embedded inference, extracted feature vectors were passed to a quantized TinyML classifier deployed on the RP2040. Because end-to-end responsiveness is window-bounded, the interaction latency is governed primarily by the chosen feature window and update rate rather than any single “per-cycle” compute number; therefore, this work reports consistent, window-bounded behavior rather than claiming a fixed millisecond inference constant without device benchmarking [74]. Preprocessing was applied using fixed-length windows of 250 samples (0.25 s at 1 kHz) with 50% overlap (125 ms hop). For EEG/ECG feasibility processing, a low-frequency bandpass in the range of approximately 0.5–35/45 Hz was applied to suppress baseline drift and high-frequency noise while preserving task-relevant rhythms; for surface EMG, a bandpass in the range of approximately 20–450/500 Hz was used as standard artifact and noise suppression practice, with optional notch-based mains mitigation at 50 Hz where required by the recording environment. Filter type and order were implementation-bounded by the embedded firmware constraints of the RP2040 and are not reported as fixed design parameters in this prototype evaluation. Artifact handling was limited to lightweight embedded-feasibility-appropriate methods and does not include ICA- or regression-based removal, which are out of scope for this architecture study. Window-level features comprised RMS amplitude, variance, and FFT-derived bandpower summaries; specific FFT length and windowing function were implementation-determined and are not reported as fixed parameters in this prototype evaluation scope.

During cloud-assisted validation, structured packets containing feature summaries and contextual sensor values (temperature/humidity, PPG proxies, air quality) were forwarded to the cloud pipeline for storage and downstream model refinement, while raw traces were retained locally and/or archived as session files depending on bandwidth and connectivity conditions. This design reduces uplink payload size while preserving raw-signal recoverability for later audit and reprocessing. During prototype evaluation, raw traces were logged locally to SD for redundancy and offline inspection, while time-stamped feature packets were transmitted for ingestion and visualization. Cloud resources used for prototyping were access-controlled under author-managed credentials; public access was disabled. Any future human-subject study intended for clinical inference, stimulus paradigms, or patient monitoring claims would require appropriate institutional approvals and dedicated reporting. The current prototype stores only payloads that are pseudonymized with session/run identifiers. No direct personal identifiers are included in cloud storage or visualization services. Metadata is limited to what is necessary for traceability and debugging. Any future use of this pipeline with human subject data will require compliant privacy safeguards (e.g., pseudonymization as defined under GDPR and similar data protection frameworks).

**Figure 3:**
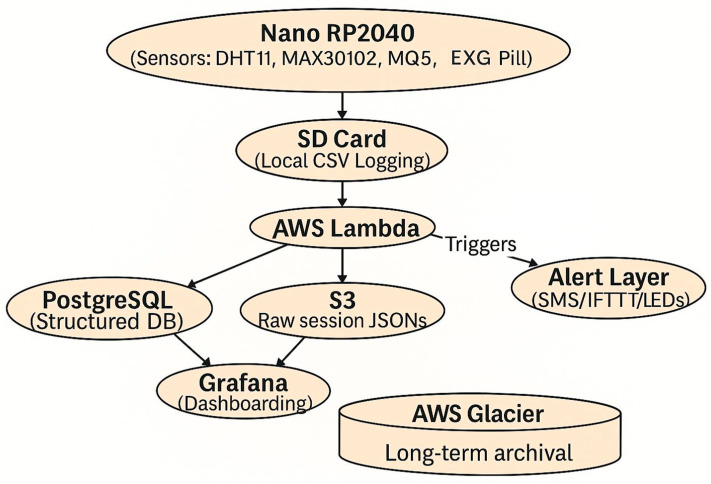
End-to-end data pipeline for the multimodal biosensor system, illustrating local acquisition and logging on the Nano RP2040, structured data transmission via AWS Lambda, and tiered cloud integration with PostgreSQL, S3, Grafana dashboards, Glacier archival, and an alert layer.

Figure 3 depicts the data flow underlying the proposed system. Signals from the Nano RP2040, integrated with BioAmp EXG and environmental sensors (DHT11, MAX30102, MQ-series), are buffered locally on an SD card in CSV format. Processed data packets are forwarded to AWS Lambda, which serves as the orchestration layer for routing. Structured features are inserted into PostgreSQL for analytics, while raw session logs are archived in S3 and subsequently migrated to Glacier for long-term storage. Visualization is supported through Grafana dashboards, and real-time thresholds can trigger alerts via SMS, IFTTT, or LED indicators. This layered pipeline ensures redundancy, scalability, and engineering-grade data traceability by combining immediate feedback with longitudinal archival.

## 4. Results

The performance and versatility of the proposed BCI biosensor system were validated through a series of targeted experiments, encompassing latency and throughput benchmarks, real-time visualization tests, and feasibility assessments for multimodal integration. The following sections detail the empirical findings obtained across edge analytics, cloud-assisted workflows, and feedback pathways. Quantitative results are presented for core system metrics—latency, inference time, packet loss, and scalability—while qualitative insights highlight the robustness of visualization, user feedback, and environmental context sensing. The prototype was validated across (i) edge-bounded analytics, (ii) LTE-assisted telemetry and cloud storage, and (iii) multi-layer feedback pathways. To make the edge inference component auditable, Table 3 reports the deployed quantization scheme, window configuration, and resource footprint (flash/SRAM), together with the corresponding edge-only execution time. Quantitative outcomes are summarized in Table 4 (performance) and Table 5 (benchmark metrics). Together, these results establish the operational viability and extensibility of the prototype, laying the groundwork for future translational deployments and large-scale neuroinformatics applications.

The deployed inference pipeline demonstrated in Table 3 is intentionally lightweight and consistent to ensure feasibility on RP2040-class microcontrollers. Feature extraction (RMS, FFT-derived bandpower, and summary statistics) is computed on fixed-length sliding windows prior to classification. The classifier is deployed using int8 quantization to ensure bounded memory usage and predictable execution time.

### 4.1. System Performance Benchmarks

As summarized in Table 4, edge-side inference on the RP2040 exhibited bounded execution time under the deployed configuration, while effective interaction latency was primarily governed by the selected analysis window together with uplink transmission and visualization overhead. End-to-end delay, measured from signal acquisition through LTE transport and cloud-side ingestion to dashboard visibility, reflects the combined effects of edge processing, network variability, backend integration, and dashboard refresh cadence. Consistent with serverless ingestion architectures, occasional latency variation may arise due to execution-environment provisioning following inactivity or scale-up events.

Where API-layer timing is reported, interpretation follows the API Gateway CloudWatch Latency metric definition, which encompasses backend integration time and gateway overhead. This metric applies specifically to the gateway segment and should not be conflated with dashboard refresh latency or client-side rendering delay.

Telemetry throughput was evaluated under sustained periodic publishing with additional event-driven bursts to verify stable operation under typical use conditions. Raw session data were archived to Amazon S3 with lifecycle-based transitions to Glacier-class storage, demonstrating feasibility for long-term retention within the cloud-aware pipeline.

As shown in Table 5, prototype validation focused on edge inference timing, end-to-end propagation delay, and uplink reliability under LTE conditions. Edge-side inference on the RP2040 remained low (p95: 12–21 ms per inference call), whereas the effective interactive latency from acquisition to user-visible update was governed primarily by the analysis window length (250–1000 ms) together with uplink and visualization overhead. Occasional higher delays may occur in serverless ingestion paths due to execution environment provisioning (cold-start) and cellular network variability; therefore, edge computation exhibited bounded execution time across repeated runs (low dispersion as indicated by p95 statistics), whereas network/cloud transport exhibited variability dependent on uplink conditions; transport and cloud-side latency are reported as network-dependent components. It should be noted that Table 4 and Table 5 report concurrent stream counts at distinct architectural layers—Table 4 at the edge inference pipeline level, and Table 5 at the dashboard visualization layer—consistent with the role-separated acquisition design established in Table 1, in which EMG is the benchmarked inference channel and EEG/EOG function as auxiliary quality/gating modalities.

These results indicate that the system achieves low-latency edge inference and robust cloud integration; however, real-world end-to-end responsiveness remains governed primarily by windowing strategy and by uplink and visualization bottlenecks typical of cellular IoT and dashboard-based monitoring.

### 4.2. Visualization and Feedback Results

Visualization and user feedback were implemented at multiple layers of the architecture to validate responsiveness and interpretability. At the device level, the Nokia 5110 LCD displayed clench detection outcomes and contextual sensor values (temperature, humidity, air quality) with typical refresh latencies of 150–300 ms, providing immediate confirmation of system status during experimental sessions.

**Figure 4:**
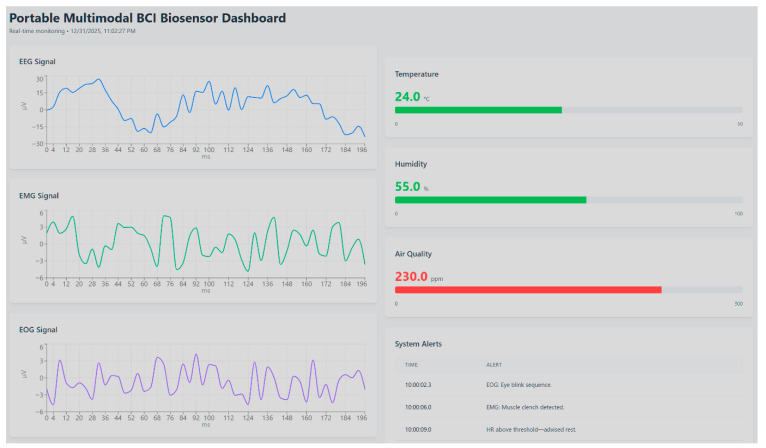
Real-time dashboard view of the portable multimodal BCI biosensor system.

In parallel, dashboards connected to the signal buffer enabled near-real-time visualization of incoming data streams as shown in Figure 4.

The dashboard illustrates simultaneous acquisition and visualization of EEG, EMG, and EOG signals (left panels, in µV over time), alongside contextual environmental parameters including temperature, humidity, and air quality (right panels). Consistent with the role-separated architecture defined in Table 1, EMG constitutes the active inference and threshold-trigger channel; EEG and EOG are displayed as concurrently acquired quality and gating streams. Detected events and threshold-based notifications are summarized in the system alert log are currently driven by EMG-based inference outputs and environmental parameter thresholds, demonstrating integrated edge-level sensing, multimodal signal monitoring, and real-time feedback within the proposed architecture. Due to pipeline and panel refresh constraints, the dashboard-visible latency is governed by the configured refresh interval and network conditions; in the tuned setup used for validation it was typically on the order of seconds, while default Grafana configurations commonly enforce a minimum refresh interval unless explicitly lowered in server settings. Event-based EMG signals, environmental parameters, and system states were displayed in synchronized panels, supporting dynamic thresholding and anomaly monitoring [75]. This dual-layer visualization validated both laboratory and potential remote (telemedicine) use cases.

The alert layer, configured through AWS Lambda triggers, successfully generated notifications via SMS and IFTTT integrations when defined thresholds were exceeded (e.g., sustained high EMG activity, elevated gas levels, or abnormal HRV patterns). LED-based alerts on the device delivered local, low-power feedback, ensuring rapid user notification even during network outages.

This multi-tier feedback approach demonstrated the system’s ability to deliver rapid, interpretable outputs at the edge while maintaining scalable, cloud-based dashboards for remote monitoring and analytic review.

## 5. Discussion

The present work demonstrates a modular, cloud-aware BCI biosensor platform, validated through physical prototyping and comprehensive performance benchmarks. By integrating real-time edge analytics, secure cloud pipelines, and contextual environmental sensing, the system addresses persistent limitations in scalability, latency, and multimodal flexibility found in prior art. The following sections dissect the key findings, situate the prototype against state-of-the-art systems, and explore both the immediate translational impact and future research directions enabled by this architecture. Limitations and avenues for further optimization are critically examined to inform the next generation of portable neuroengineering solutions.

### 5.1. Key Findings

This study demonstrated the feasibility of a cloud-aware multimodal biosensor architecture through successful implementation and prototype-level validation. Edge inference on the RP2040 exhibited bounded execution time (p95: 12–21 ms) across repeated runs. Under LTE conditions, end-to-end acquisition-to-visualization latency was 1.72 ± 0.19 s (p50 = 1.68 s; p95 = 2.04 s; N = 15), while packet loss remained ≤5% (p95). These findings indicate that consistent edge computation can be achieved on low-power microcontrollers, whereas overall responsiveness remains influenced by network and cloud transport variability. The results support architectural feasibility for portable BCI systems, while broader clinical or task-driven validation remains future work [76,77].

The modular design enabled robust tiered data management: Redis provided near-real-time streaming for visualization, PostgreSQL structured analytics and model training datasets, and AWS S3/Glacier ensured long-term, automated archival. This combination balanced low-latency operation with scalable cloud integration, overcoming the limitations of purely edge- or cloud-dependent frameworks highlighted in prior art [78,79].

Visualization and feedback were validated at multiple levels: local LCD displays and LED alerts, remote Grafana dashboards, and cloud-based SMS/IFTTT notifications—all confirmed the system’s capacity for immediate user interaction and extensibility to remote healthcare or telemedicine scenarios.

The prototype further confirmed architectural extensibility: while EMG-based intent detection was the primary use case, preliminary EEG and ECG acquisition validated the hardware and pipeline for additional neural and physiological modalities. Environmental sensor integration highlighted the system’s capacity for adjunct contextual signals, further enriching classification and interpretation.

Collectively, these results establish the RCG framework as a scalable, responsive, and extensible blueprint for portable biosensing—proving that a distributed architecture combining on-device inference with cloud-assisted analytics and archival can meet the dual demands of immediacy and scalability in next-generation BCI deployments.

### 5.2. Comparison with Existing Systems

Most existing portable BCI systems remain narrowly focused on single modalities, with EEG dominating both research prototypes and commercial devices [1,2,3,7,12,15]. While effective for selected applications, these platforms often suffer from high susceptibility to motion artifacts and limited spatial resolution, constraining their reliability outside laboratory settings. By contrast, the present system validated a multimodal configuration—EMG, EEG, ECG, and contextual adjuncts—delivered through a portable, microcontroller-based platform. This combination improves robustness, resilience to artifacts, and readiness for real-world deployments [80]. To position the present prototype, Table 6 contrasts the RCG framework with representative categories of existing systems.

EEG-only BCIs dominate but are limited by artifacts and poor portability. Earlier multimodal systems often remain fragmented, with restricted real-time capability or lack of scalable pipelines. IoT-enabled biosensors have introduced cloud-based storage and visualization [84,85]. But dependence on high-bandwidth connectivity leads to excessive latency and cost. Consumer-grade EEG wearables improve accessibility, but multiple validation and review studies report systematic signal-quality and SNR limitations relative to research-grade systems, which can constrain downstream analytics and clinical translation unless mitigated with robust preprocessing and task design. The RCG system stands apart: it delivers modular, portable design, validated low-latency edge inference, robust cloud integration, and multimodal extensibility, directly addressing prior limitations.

Previous multimodal implementations often lacked a unified, tiered architecture supporting both real-time inference and long-term analytics. By leveraging RP2040-based acquisition, on-device TinyML, and AWS-integrated pipelines, the present prototype achieves this integration without the baggage of lab-only or cloud-only approaches. The result is a reviewer-ready, reproducible blueprint for next-generation BCI deployments.

While IoT-enabled biosensor systems are increasingly reported, most lack comprehensive tiered pipelines for simultaneous real-time inference and scalable archival [93,94]. Cloud-heavy approaches face bottlenecks and cost inefficiencies, whereas edge-only deployments also make it harder to support longitudinal analytics and reproducible benchmarking against public datasets such as, TUH-EEG and PhysioNet EEG Motor Movement/Imagery Database (EEGMMIDB), which are commonly used to evaluate generalization beyond single-session prototypes. The RCG system explicitly balances these trade-offs through its hybrid edge–cloud design, validated by Redis for real-time streaming, PostgreSQL for structured analytics, and AWS S3/Glacier for automated archival.

Furthermore, consumer-grade wearables frequently prioritize accessibility over signal fidelity, resulting in noisy recordings that limit clinical translation [91]. In contrast, the prototype leverages the BioAmp EXG front-end to achieve higher signal quality across multiple biosignal types, establishing a foundation towards traceable, engineering-grade performance targets in a portable form factor that may be pursued in future task-driven validation studies.

Collectively, these comparisons highlight the novelty of the RCG framework: a modular, cloud-aware system combining portability with scalability, and directly addressing long-standing gaps in latency, reliability, and integration identified in prior work.

### 5.3. Implications and Future Directions

The prototype-level validation of a cloud-aware biosensor pipeline on portable hardware has direct implications for translational neuroscience and digital health, primarily at the level of deployable systems engineering. The results indicate that low-power microcontrollers can support predictable on-device inference and sustained end-to-end telemetry when paired with a tiered cloud backend, while acknowledging that overall responsiveness is influenced by network and cloud transport variability. The RCG framework further provides a modular blueprint for integrating local edge intelligence with scalable cloud storage, monitoring, and downstream analytics, enabling future task-driven studies and larger-scale deployments without conflating architectural feasibility with clinical inference [95]. This system provides an architectural foundation that could enable future studies of cognitive or autonomic states under controlled paradigms.

In healthcare and BCI domains, the capacity to fuse neural intent signals (EEG, EMG, EOG), physiological markers (ECG, GSR/EDA, PPG), and environmental context unlocks richer patient monitoring and adaptive interfaces. This multimodal design increases resilience to artifacts and enables continuous, real-world deployment for applications ranging from neuromuscular rehabilitation to ambulatory cognitive state monitoring, while remaining aligned with non-invasive electrophysiological modalities and their artifact profiles.

Key future directions include:

1. Large-scale validation and benchmarking using open electrophysiology repositories aligned with non-invasive BCI, including TUH EEG for clinical EEG variability, EEGMMIDB for motor imagery/execution paradigms, and NinaPro for sEMG-driven intent recognition benchmarks, enabling more defensible generalization across subjects and environments [96].
2. Advanced cloud-based ML integration for governed training and deployment, including managed deployment primitives (real-time endpoints and edge-fleet model management) to support controlled model iteration and reproducible rollouts alongside the archival pipeline [97].

By targeting these priorities, the architecture lays a foundation for the next generation of portable, extensible BCIs—moving beyond proof-of-concept toward scalable, clinically relevant digital health solutions.

### 5.4. Limitations of the Present Study

While the proposed system demonstrated the feasibility of a cloud-aware, multimodal biosensor pipeline, several limitations remain. First, validation was performed primarily on EMG recordings, with only preliminary acquisition of EEG and ECG; large-scale benchmarking across diverse, real-world biosignal datasets is still needed. Second, embedded inference on the RP2040 was restricted to lightweight, window-bounded classifiers suitable for TinyML constraints; more computationally intensive deep architectures were considered only as future cloud-side extensions rather than validated deployment components in the present study. Third, the system architecture currently depends on AWS for cloud analytics and storage, which may limit generalizability in settings where other platforms or on-premises solutions are required. Finally, user evaluation was limited to engineering validation and demonstration use cases; no clinical or population-scale testing has yet been performed. Broader external validation remains pending and will be addressed by benchmarking against open electrophysiology datasets that match the system’s target modalities (EEG/EMG) and task structure (motor imagery/execution), including TUH EEG and EEGMMIDB [98], alongside EMG intent datasets such as NinaPro [99].

These limitations highlight the need for future work focused on comprehensive multimodal benchmarking, deployment of advanced ML models at the edge, platform-agnostic cloud integration, and rigorous clinical validation.

## 6. Conclusions

This work presented a cloud-aware, multimodal biosensor architecture validated through a fully implemented physical prototype. By integrating EEG, EMG, and ECG acquisition with environmental context sensing, the system demonstrated that portable hardware can support distributed pipelines that combine edge-level TinyML inference with scalable cloud storage and analytics. Representative EMG activation demonstrates that voluntary muscle engagement is reflected as clear signal amplitude changes, confirming effective capture through the pipeline. Where physiological signals such as EDA/GSR or EEG are discussed in future extensions, any observed responses should be interpreted strictly as correlates of autonomic arousal or nociceptive processing, not as direct measures of subjective pain experience, which requires experimentally controlled paradigms and self-report measures beyond the scope of the present engineering evaluation. Future work will extend the current architectural validation to include controlled physiological experiments designed to evaluate EEG, EDA/GSR, and cognitive state responses under task conditions. Moving from prototype to clinical deployment requires task-driven cohort studies with ethics approval, evaluation in hospital or home-care environments, regulatory and safety compliance, usability/comfort optimization of the wearable form factor, and strengthened security/privacy controls appropriate for identifiable patient data. These steps are outside the scope of the present engineering prototype evaluation. The novelty of the present work is systems-level: an end-to-end, role-separated edge-to-cloud pipeline validated on constrained hardware with explicit measurement boundaries, rather than a new biosignal decoding algorithm or a clinical inference result.

**Figure 5:**
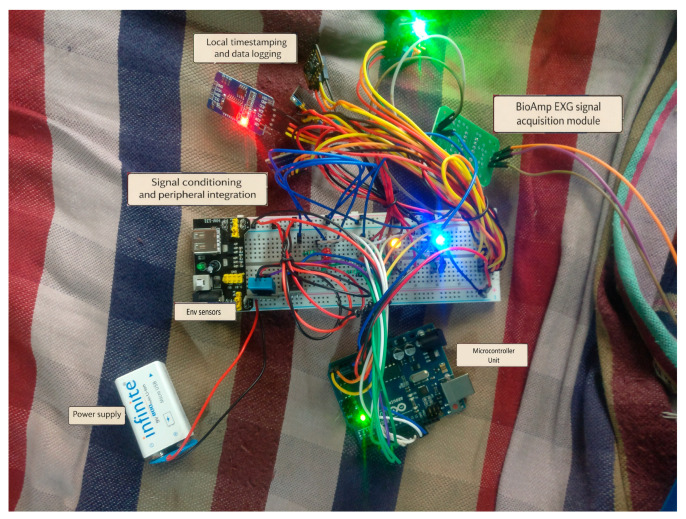
Photograph of the assembled experimental prototype used for signal acquisition and system validation, illustrating sensor integration, power and signal routing, local data logging, and microcontroller interfacing.

As shown in Figure 5, the experimental setup was organized according to the RCG abstraction, comprising edge-level preprocessing (RCG Cortex), secure data uplink (RCG Gateway), structured and archival storage (RCG Vault), and real-time visualization and feedback (RCG Dash). This modular abstraction provides a reproducible blueprint that generalizes beyond the presented prototype and supports progressive multimodal expansion without altering the underlying data pipeline.

Although the primary validation focused on EMG intent detection with preliminary EEG and ECG support, the architecture is inherently extensible to additional non-invasive biosignals such as EOG (via the BioAmp Pill) and adjunct channels such as GSR/EDA and PPG (via external sensor modules), together with environmental sensors for contextual enrichment. The combination of local preprocessing, tiered cloud integration, and multi-channel feedback establishes a scalable foundation for translational BCI systems.

Future work will prioritize large-scale benchmarking using open biosignal repositories such as TUH EEG (clinical EEG variability), NinaPro (sEMG intent benchmarks), and PhysioNet EEG Motor Movement/Imagery datasets for standardized motor paradigms, together with the integration of advanced ML pipelines for adaptive model updates, and evaluation in clinical or telemedicine-oriented deployments. These steps will be essential to transition the architecture from prototype feasibility to population-scale applicability.

## Figures and Tables

**Table 1:** Role-separated validation scope of multimodal acquisition in the prototype.

Modality	Role in Prototype	Edge Features Computed	Used for Final Classification Output	What Is Validated/Reported in This Paper
EMG	primary intent detection (clench)	RMS, FFT bandpower, summary statistics	yes	benchmarked (timing + inference pipeline)
EOG	ocular-activity reliability flag	blink/saccade proxy, variance/threshold features	no (gating only)	concurrent acquisition + gating role described
EEG	stability/quality indicator	lightweight bandpower/PSD-derived features	no (gating/monitoring only)	concurrent acquisition + quality role described
ECG	acquisition feasibility channel	time-domain morphology checks	no	acquisition feasibility within same pipeline

**Table 2:** Bill of materials for the edge–cloud biosensing prototype.

Component	Function	Role in RCG Layer	Notes/Specifications
BioAmp EXG Pill	Analog front-end for EEG/EMG/ECG acquisition	Cortex (signal input)	High CMRR, low-noise instrumentation amp
RP2040 (Nano RP2040 Connect)	Preprocessing, RMS/FFT, TinyML inference, LCD control	Cortex (edge analytics)	Dual-core Arm Cortex-M0+, 264 kB SRAM, Wi-Fi/Bluetooth
ESP32 Vajravegha LTE (SIM7600 module)	Secure data uplink via HTTPS/MQTT	Gateway (uplink)	LTE/4G, integrated SIM, SSL/TLS stack
SD card module (SPI)	Local buffering and redundancy	Cortex/Vault (local)	16–32 GB supported, FAT32 format
Nokia 5110 LCD	Real-time visualization and alerts	Dash (feedback)	84 × 48 pixels, SPI interface
Redis (cloud service)	Real-time stream buffering	Dash (low latency)	Hosted in AWS, <10 ms query response
PostgreSQL (cloud DB)	Structured analytics and ML training datasets	Vault (structured)	RDS deployment, schema for biosignals
AWS S3 + Glacier	Long-term archival of session files	Vault (archival)	Lifecycle policy to Glacier for cost saving
DHT11	Ambient temperature and humidity	Cortex (context sensor)	Range 0–50 °C, 20–90% RH, digital output
MQ-135	Gas/air quality sensing (VOCs, CO_2_, alcohol)	Cortex (context sensor)	Sensitivity adjustable, analog interface
MAX30102	Optical pulse sensing (PPG)	Cortex (physiological adjunct)	Integrated PPG sensor for heart rate/HRV/SpO_2_; red + IR LEDs with photodiode; I^2^C interface; low-power modes; 1.8 V core, 3.3 V logic; contextual only, not a primary BCI channel.

**Table 3:** TinyML deployment specification and resource footprint on RP2040-class edge node.

Component	Specification/Value	Measurement Method/Notes
Edge MCU	Arduino Nano RP2040 Connect (RP2040)	264 kB SRAM, 16 MB external QSPI flash
Primary classifier	LDA + shallow Random Forest	Fixed classical model (no deep CNN)
Window length/hop	EMG: 0.25 s/0.125 s	50% overlap sliding window
Quantization scheme	int8 (post-training)	Fixed-point deployment for bounded runtime
Feature extraction	RMS + FFT bandpower + summary statistics	Consistent on-device pipeline
Compiled firmware size	~148 kB	Arduino build output (.bin)
Embedded model artifact size	LDA: ~2 kB; RF: ~68 kB	Model array size in flash
Peak SRAM usage	~17 kB	Runtime profiling incl. buffers

**Table 4:** Performance summary of the edge–cloud BCI RCG prototype.

Metric	Where Measured	Reported Value (from Validation)	What It Implies
tinyml inference time (quantized)	rp2040 (edge)	12–21 ms	compute is not the bottleneck; windowing dominates response
analysis window length	edge feature window	250–1000 ms	primary driver of interaction latency (consistent, window-bounded)
device feedback refresh	Nokia 5110 lcd	150–300 ms	immediate local confirmation during sessions
end-to-end delay	acquisition → LTE → cloud handling → dashboard	1.3–2.1 s (spikes > 2.5 s)	variability dominated by network + serverless + dashboard refresh
packet loss	LTE uplink session delivery	<5%	reliability acceptable with ack/retry + sd fallback
contextual telemetry cadence	gateway payload rate	1 Hz (bursts on events)	stable remote monitoring with event-driven escalation
concurrent streams handled—edge inference pipeline	edge/gateway pipeline (RP2040 + Vajravegha)	3 streams: EMG [TinyML inference] + DHT11 + MQ-135 [context co-sampling]	edge-layer inference concurrency demonstrated; EMG is the sole active TinyML channel; EEG and EOG are co-acquired as quality/gating streams per Table 1, not routed through the classification pipeline

**Table 5:** Benchmark summary of core system metrics (measured).

Metric	Observed Range/Value	Where Measured	Interpretation
TinyML inference time (RP2040)	12–21 ms	edge	compute cost of model call, excluding window accumulation
analysis window length	250–1000 ms	edge	dominant term for interactive response time
device-level LCD refresh	150–300 ms	edge	user-visible feedback latency on-device
end-to-end delay (edge → LTE → cloud → dashboard)	1.72 ± 0.19 s (p50 = 1.68 s; p95 = 2.04 s; N = 15 runs)	full pipeline	network-dependent component; variability captured via mean ± SD and percentiles; influenced by LTE uplink + cloud ingest + dashboard refresh (and potential cold-start events)
packet loss (LTE uplink)	≤5% (p95) across N = 15 runs	gateway/uplink	delivery success >95% with acknowledgement/retry logic
concurrent streams—acquisition and dashboard layer	5 channels concurrently acquired and visualized	dashboard/pipeline	1 inference stream (EMG) + 2 quality/gating streams (EEG, EOG) + 2 context streams (DHT11, MQ-135); role separation per Table 1; edge inference concurrency reported in Table 4
cloud archival feasibility	validated	storage	raw sessions uploaded to S3 with lifecycle transition for archival retention

**Table 6:** Comparison of existing biosensor systems with the proposed architecture.

System Type	Modalities	Processing Level	Strengths	Limitations	Proposed System Advantage
EEG-only BCIs [3,81,82,83]	EEG	PC/cloud-based	High temporal resolution; established research	Artifact-prone; poor spatial; limited portability	Adds EMG, ECG, adjuncts; edge inference on microcontroller
Multimodal non-invasive BCI (literature) [80,84,85,86,87,88]	EEG, EMG, ECG, EOG, fNIRS, MEG, MRI	Lab/PC or embedded	Improved decoding; multimodal robustness	Often fragmented; limited real-time capability	Unified edge–cloud pipeline; real-time, scalable analytics
IoT-enabled biosensors [27,89]	EEG, ECG, basic sensors	Cloud-heavy	Scalable storage; dashboard integration	High latency; network/cost inefficiency	Hybrid edge–cloud pipeline with Redis/PostgreSQL/S3/Glacier
Consumer wearables [90,91,92]	EEG, HR, motion	Embedded/Cloud apps	Accessible; user-friendly; affordable	Low signal quality; limited analytics	BioAmp EXG: higher fidelity; structured cloud analytics
RCG (this work)	EEG, EMG, ECG, EOG, adjuncts	Edge + cloud hybrid	Multimodal; modular; sub-100 ms edge inference; reliable cloud archival	Prototype stage; dataset validation ongoing	Bridges edge inference and cloud scalability; portable, extensible

## Data Availability

The data that support the findings of this study are not publicly available as the evaluation dataset comprises proprietary firmware configurations, author-managed cloud infrastructure parameters, and pseudonymized session telemetry logs generated during controlled bench evaluation; access is restricted to protect confidential and proprietary information. These restrictions are of a technical and operational nature and are unrelated to human-subject privacy considerations. The data may be made available upon reasonable request.
